# Growing Trend of Fighting Infections in Aquaculture Environment—Opportunities and Challenges of Phage Therapy

**DOI:** 10.3390/antibiotics9060301

**Published:** 2020-06-04

**Authors:** Justyna D. Kowalska, Joanna Kazimierczak, Patrycja M. Sowińska, Ewelina A. Wójcik, Andrzej K. Siwicki, Jarosław Dastych

**Affiliations:** 1 Proteon Pharmaceuticals, 90-364 Lodz, Poland; jkazimierczak@proteonpharma.com (J.K.); psowinska@proteonpharma.com (P.M.S.); ewojcik@proteonpharma.com (E.A.W.); jdastych@proteonpharma.com (J.D.); 2 Department of Microbiology and Clinical Immunology, Faculty of Veterinary Medicine, University of Warmia and Mazury, 10-719 Olsztyn, Poland; siwicki@uwm.edu.pl

**Keywords:** phage therapy, aquaculture, antibiotic resistance, phage, pathogenic bacteria

## Abstract

Phage therapy, a promising alternative to antimicrobial treatment of bacterial diseases, is getting more and more popular, especially due to the rising awareness of antibiotic resistance and restrictions in antibiotics’ use. During recent years, we observed a growing trend of bacteriophages’ application in aquaculture, which in each year reports high losses due to bacterial diseases. This review provides an update of the status of bacteriophage therapy for the treatment and prevention of infections in the aquatic environment. As it is still mostly in the scientific stage, there are a few constraints that may prevent effective therapy. Therefore, specific characteristics of bacteriophages, that can act in favor or against their successful use in treatment, were described. We underlined aspects that need to be considered: specificity of phages, bacterial resistance, safety, immune response of the host organism, formulation, administration and stability of phage preparations as well as bacteriophages’ influence on the environment. The biggest challenge to overcome is finding the right balance between the desired and problematic characteristics of bacteriophages. Finally, regulatory approval challenges may be encountered by bacteriophage manufacturers. Even though there are still some technical constraints connected with the global use of bacteriophage therapy, it was concluded that it can be successfully applied in aquaculture.

## 1. Introduction

Over the last 50 years, the demand for aquaculture production has been significantly growing. The biggest producers are Asian countries that are responsible for 89% by volume and 79% by value of global production [1]. According to the FAO report from 2018, the total first sale value of fisheries and aquaculture production in 2016 was estimated at USD 362 billion, of which USD 232 billion was from aquaculture production [2]. However, there are factors limiting this growth such as animal diseases. They have a big economic impact on aquaculture, causing vast losses. It is estimated that 34% of infections are of bacterial origin [3].

A growing production in aquaculture is a direct response to the high consumption of fish and seafood. Therefore, diseases attacking these animals have tremendous impact on public health as pathogens can be spread through direct contact with sick animals or animal-source food. The majority of zoonotic diseases can be easily cured with appropriate therapy, however, some zoonoses, if left untreated, can cause systemic diseases that can lead to death of the patient or cause public health risks [4].

An additional threat comes from the antimicrobial resistance of pathogens. This is partly impacted by the use of antibiotics in animal production as it is estimated that farm animals consume 73% of all antibiotics in the world [5]. Nowadays, they are used not only to treat serious infections, but also as preventive agents or growth promotors. As early as the 1940s, it was proved that the amount of produced meat can be increased by treating animals with broad-spectrum antibiotics [6]. Even though antibiotic resistance was rising among farm animals, they were used continuously as growth promoting agents. Thus, nowadays the diseases that were previously easily cured are becoming a major problem, as a lot of bacteria are multidrug-resistant [7]. Moreover, in some cases, even when antibiotic therapy is effective in laboratory trials, its efficacy in vivo may be very low [8].

Another important aspect is the fact that in 2006, the EU authority introduced a ban for antibiotic usage as growth promoters (AGPs) in animal production. Moreover, there is a new EU legislation, which is expected to become law by 2022, which bans the use of antibiotics that are important for human medicine and prohibits the use of any antimicrobials in livestock without a prescription from a vet. Thus, antimicrobials could no longer be used to improve the performance or compensate for poor animal husbandry and it also applies to single animals—not only herds or flocks.

Therefore, scientists all over the world are working on the development of alternative treatments. Bacteriophage research is a dynamically developing field with promising prospects, and beside some challenges, there is an observed rise in interest about phage therapy in aquaculture [9,10,11,12,13].

In the presented paper, we suggest a discussion over some specific characteristics of bacteriophages on the example of aquaculture. It was noticed that their unique features can be used in favor or against effective phage therapy, depending on the application.

## 2. Bacteriophage Overview

Bacteriophages (also known as phages) are defined as viruses that infect bacteria [14,15]. Since their discovery at the beginning of the 20th century by Frederick Twort and Felix d’Hérelle, independently, phages were designed for therapeutic use. However, they never became everyday treatments because of the rising popularity of antibiotics in those days, although the research on phages was continued in the former Soviet Union (Georgia and Russia) and Poland [14].

The majority of known phages are tailed viruses which belong to the *Caudovirales* order. They are built of a capsid with nucleic acid (either DNA or RNA) and of a tail that may vary in size. The capsid consists of proteins or lipoproteins that protect the genetic material of the phage, while the tail recognizes the bacterial host via specific receptors located at the tail fibres [11,14]. In aquaculture, phages of different families can be encountered for phage therapy, mostly *Siphoviridae*, *Myoviridae* or *Podoviridae* [16,17,18,19,20].

Phages can exhibit two distinct replication cycles: lytic and lysogenic. Viruses showing only a lytic mechanism of replication are called virulent, while the ones exhibiting both lytic and lysogenic cycles are temperate phages. In the lytic cycle, bacterial cells are directly lysed as a result of a viral infection. The lysogenic cycle is defined when a host cell is not destroyed immediately, like in the case of virulent phages, but when the phage genome is inserted in the form of prophage into the bacterial chromosome and is replicated together with its host genome. Alternatively, prophages can exist in host cells in the form of plasmids. This state can last for many generations until the introduction of a lytic cycle induced by a certain stress factor (e.g., antibiotic treatment, DNA damage, etc.) [11,14].

Temperate phages (those exhibiting lysogenic life cycle) are considered as the ones taking part in a horizontal gene transfer (HGT) between bacterial cells. They can transfer virulence factors orantibiotic resistance genes and therefore they are not suitable for therapy. On the contrary, virulent phages lyse the host cells directly and their possibility to transfer any genes is limited, which make them desirable for therapeutic purposes [11,15].

## 3. Opportunities and Challenges of Bacteriophage Therapy

Bacteriophage therapy applied in food-producing animals might be an important alternative to antibiotics, especially in aquaculture production. Aquaculture belongs to those branches of animal production that use very large quantities of antibiotics, which, in addition to problems with increasing antibiotic resistance among bacteria, is a direct threat to the aquatic environment. The accumulation of antibiotics in bottom sediments leads to the destruction of not only target bacteria but also other microorganisms and disturbs the ecological balance of the aquatic environment. Phages act directly only on targeted bacteria which allows to fight aquaculture bacterial pathogens and reduce the spread of food-borne diseases in humans [21,22]. What is more, as self-replicating and self-limiting entities, phages minimize the risk of environment contamination. Considering the wide range of pathogens and high level of specificity of bacteriophages, the most practical approach in phage therapy is the use of cocktails of phages with a different specificity spectrum. As more and more phages are identified and characterized, the simultaneous treatment of different bacterial pathogens is possible [13,23]. It was also noticed, that a single administration of phages is not as effective as repeated treatment or continuous administration [12]. To date, several phage preparations have already been described, the use of which in medicine and agriculture has proved to be successful, and some of them are already on the market: A phage cocktail composed of 12 phages on open wounds (“Phagoburn”) [24], BioPhage-PA-cocktail on nosocomial infections [25], AgriPhage^TM^ preparation to protect crops [26] or LISTEX to treat food products against *Listeria monocytogenes* [27].

Bacteriophage therapy in aquaculture has already been proved successful in multiple experimental reports. The conducted literature research showed that most of the experimental in vivo phage therapies focus on *Vibrio* species (16 out of 24), but also *Aeromonas*, *Pseudomonas*, *Acetobacter* and *Flavobacterium* have been addressed (Table S1).

The studies on aquaculture showed an overall protective effect of phage therapy on fish and shellfish, thus providing an optimistic outlook on future benefits of phage-based technologies for treating diseases in aquaculture. However, the therapeutic effect on the veterinary market is affected by proper diagnostics as well as animal conditions such as age, health or stress factors. For example, under- or overfed animals may not respond to bacteriophage therapy. Therefore, monitoring for specific pathogens will help identify the environmental risks and undertake the correct actions [12].

Bacteriophage therapy for aquaculture is still mostly in the scientific stage and needs to be further studied and described [15]. It is already evident that it can be applied to prevent and treat infectious diseases and it was proved in some cases to be even more effective than antibiotic treatment [11]. However, there are still a few major constraints that may prevent the successful use of phage therapy in current treatment.

Specific bacteriophage characteristics may act in favor or against a particular application. Some characteristics of bacteriophages that are usually seen as advantages (for example narrow host range) can become drawbacks when designing phage therapy and should be well thought out. In Table 1, we present a few crucial aspects of phage research that are considered and further discussed in the article from both sides: opportunities and challenges.

### 3.1. Specificity of Phages

There is a general consideration that phages have little side effects compared with antibiotics because of their narrow specificity. Phages’ actions are limited only to a few or several bacterial strains. Due to the fact that bacteriophages recognize receptors on the target bacteria surface, they are highly specific, infecting only one bacterial genus or even certain particular strains. Bacteriophages specific to pathogens do not harm natural and beneficial microflora of the environment or the host organism [11]. Moreover, they do not interact with eukaryotic cells but target only bacteria, therefore no adverse effects were observed in the reported trails [14,28]. In 2016, Silva et al. applied a phage therapy for *Aeromonas salmonicida* infecting *Solea senegalensis*. They observed that bacteriophages had no influence on natural bacterial communities of aquaculture water, while at the same time, lowered the *Aeromonas* infection [29]. In contrast, antibiotics, particularly those with a wide activity spectrum, alter the normal environment and host microflora. They may cause secondary infections and colonization of resistant and opportunistic pathogens [30]. In the case of aquaculture, the administration of antimicrobials is mostly to water, with or without feed. It results in an environmental disturbance of the commensal bacteria, and can cause a spread of the antibiotics to neighboring ecosystems [31].

However, when different or genetically unrelated strains are to be treated, the narrow host range of bacteriophages can become a drawback [26]. Therefore, phages that cover a wide spectrum of bacteria are usually in the area of research interest [32]. Although it is rare to find phages with a broad lytic activity, there are examples of virulent phages in aquaculture that seem to be promising for therapeutic use. For example, Vinod et al. were able to isolate a phage active against 50 isolates of *V. harveyi* to protect shrimp larvae [33], and Zhang et al. found a phage with a broad host range against *V. parahaemolyticus* [19]. As such case is not so frequent, a solution to overcome the challenge of a narrow host range is to use phage cocktails composed of a few phages that all together ensure a broad host range, but still do not affect favourable microflora. For example, Imbeault et al. showed that phages have an additive effect when protecting fish farms from *Aeromonas salmonicida* [34], and Kalatzis et al. proved the desired effect of the combination of two lytic phages against *Vibrio alginolyticus* [35]. There was also a study in which a bacteriophage cocktail BAFADOR^®^ (containing bacteriophages against *Aeromonas hydrophila* and *Pseudomonas fluorescens*) was used by Schulz et al. to test the survival of European eel [36]. In another experiment, Stalin and Srinivasan focused on the selection of the most efficient phages to the cocktail against *Vibrio harveyi* in shrimp aquaculture [37]. Moreover, as shown by Kokkari et al., sometimes a narrow host range of a phage is not a limiting factor for its usage in a therapy. There are phages not suitable for universal therapy but if they are isolated from a host causing a recurrent infection, they can be considered as an alternative for other forms of treatment, as in the case of the VEN phage [38].

### 3.2. Bacterial Resistance and Other Safety Aspects

A major concern nowadays in both human and veterinary medicine is the development of antimicrobial-resistant bacterial strains (AMR). Intensive aquaculture and hence use of antibiotics on a mass-scale in past decades has led to the low efficacy of such a treatment and to the accumulation of these therapeutics in the environment leading to resistance of pathogens.

Bacteriophages come as a natural candidate to solve this problem [38]. Their mechanism of action is different than antibiotics, therefore they can act even on multidrug-resistant strains, making them very advantageous for use in therapy. There are multiple cases in which phages were used when no other treatment was effective. One of the examples can be the treatment of multidrug-resistant *Aeromonas salmonicida* subsp. *salmonicida* with the virulent phage ASP-1. It was shown that this phage would be promising as a component of a phage cocktail and can act as a potential biocontrol agent in the environment [39]. In another study by Hoang et al., phages against antibiotic-resistant *A. hydrophila* were isolated and characterized. It was reported that in Asian countries, the resistance of this pathogen can reach up to 93%–100% to commonly used antibiotics. Furthermore, another negative effect of antibiotics is that their residuals are present in the body of the animal even after therapy is finished, contrary to phages which are present only in the presence of pathogenic bacteria. It was reported that from 2014 to 2016, Vietnamese producers were very often rejected by other markets because of the exceeded level of antibiotic traces found in fish, leading to high economic losses. Therefore, preliminary studies were conducted in which a cocktail of two phages active against *A. hydrophila* was composed [40].

Moreover, a very common problem is the development of bacterial biofilms which enable bacteria to develop even higher antibiotic resistance than in the planktonic state. In the case of aquaculture, *Vibrio* sp. is able to form biofilms which are linked to its pathogenicity. Fortunately, phages are known to be effective against bacterial biofilms which are very difficult targets for most antibiotics. According to Kim et al., the bacteriophage pVa-21 was able to disperse *V. alginolyticus*’s biofilm and was considered promising in phage therapy [41].

On the other hand, one of the challenges encountered by phage therapy is the development of bacterial insensitive mutants, which are resistant to applied bacteriophages [26]. It is supposed that using bacteriophages on a great scale in the future may result in different side effects. Nevertheless, even though bacteria can develop different mechanisms of phage resistance, bacteriophages continuously adapt to different conditions to overcome these obstacles. The tendency of bacteria to develop resistance is about ten times slower than in the case of antibiotics, and phage-resistant bacteria are not resistant to other phages having a similar target range [42].

There are at least a few described mechanisms of bacterial resistance to viruses including the following: blocking phage adsorption, preventing DNA injection, cutting a phage’s genetic material, inhibiting the replication of phage DNA or assembly of phages and suicide of bacteria [43,44]. One of the most popular is the mutation of cell surface receptors so that phages cannot absorb the bacterium, hence they do not initiate infection. Bacteria can delete certain proteins, overproduce, e.g., capsular polysaccharides or immunoglobulin G-binding protein A *(Staphylococcus aureus*) as well as change the conformation of outer proteins (Gram-negative bacteria) for this purpose. However, phages can also modify their receptor binding proteins and thus be able again to attach to the bacterial surface [26,44,45,46]. Next, bacteria can prevent DNA injection, even if a phage is successfully attached to its surface, by the superinfection exclusion system (Sie), thus providing immunity to strains containing prophages [47]. Another strategy of resistance—cleavage of phage DNA—includes the use of a restriction–modification system that degrades the nucleic acid of a phage or the development of adaptive immunity through interfering with clustered regularly interspaced short palindromic repeats (CRISPR) sequences. In the case of a restriction–modification system, unmethylated phage DNA is cut by a restriction enzyme, however, very often phages do not have recognition sites for endonucleases and can escape bacterial resistance this way. The CRISPR-Cas system, present in about 40% of sequenced bacteria, is more complicated and involves integrating spacers (small fragments of foreign DNA) into CRISPR loci that target phage nucleic acids, enabling the Cas nuclease protein to cleave them. Again, phages adapt mechanisms to overcome this system by point mutations in spacer sequences or the PAM region [42,43,44,45]. The replication of phage DNA can be blocked by the bacteriophage exclusion (BREX) system, encoded by a few genes aiming at the prevention of the first round of the bacteriophage lifecycle [44]. Another possibility used by some bacteria (e.g., *S.aureus* or *V.cholerae*) is blocking the phage assembly by phage-inducible chromosomal islands (PICI), leading to producing capsids with bacterial instead of phage DNA [44]. Bacteria develop also abortive infection systems (Abi) of which the best known is the Rex system found in λ-lysogenic *E. coli* strains, however, they are also found among aquaculture pathogens, especially *Vibrio*. It involves proteins interacting with phage DNA, leading to a drop in the ATP level and thus preventing phage multiplication. This system acts usually against phage replication, transcription or translation [43,47]. Moreover, Cairns et al. demonstrated that simultaneous exposure to an antibiotic and a phage increased the adaptation against both of the stressors. The same researchers in 2017 observed that while the presence of a phage did not affect the antibiotic resistance, the presence of an antibiotic affected the phage resistance [48].

Therefore, the application of bacteriophage cocktails or combinations of bacteriophages with other antimicrobials seems to be a successful way to avoid bacterial resistance because developing resistance to more than one phage would mean a higher fitness cost for the bacteria [47,49]. There are multiple examples in which this issue is successfully addressed. For example, Mateus et al. described an efficient use of a cocktail composed of three bacteriophages, active against *Vibrio* in aquaculture, which might delay the development of phage resistance [50]. Moreover, Gu et al. used a cocktail of three phages against *K. pneumoniae* and observed a lower number of phage-resistant mutants than in the case of a single phage [51]. Next, O’Flynn et al. studied *E. coli* phages and noticed that phage cocktails resulted in a lower resistance frequency in bacterial strains [52]. All in all, these results strongly confirm the reliability of the use of phage cocktails because then problems with phage resistance in aquaculture might be avoided.

It is worth to mention that phage-resistant bacteria are not always associated with negative effects. They frequently exhibit a decline in bacterial virulence because of losing the ability for colonization and increasing sensitivity to other phages [47]. Laanto et al. showed *Flavobacterium columnare* modulating virulence after phage-caused phenotypic changes in bacteria grown outside of the fish host [53]. Carrillo et al. demonstrated *Campylobacter* isolates which significantly reduced the ability to colonize the broiler intestine compared with the original susceptible strain [54]. Further, Filippov et al. proved that the phage-resistant *Yersinia pestis* was less virulent because of modifications in lipopolisacharides (LPS), and Lin et al. showed similar effects in *Klebsiella pneumoniae* when the K1 capsule, being a phage receptor, was modified [55,56]. Moreover, there was a study in which a phage-resistant *V. anguillarum* strain lost its virulence, which was notified by the reduced mortality of cod [57]. Chan et al. showed a phage resistance management strategy that allows to take over the control on multidrug-resistant *Pseudomonas aeruginosa*. They proved that the phage that utilizes the outer membrane porin M (OprM) of the multidrug efflux systems MexAB and MexXY as a receptor-binding site, when promoting phage resistance in bacteria, changes the efflux pump mechanism at the same time, causing increased sensitivity to drugs from several antibiotic classes [58]. That strategy represents a new approach to phage therapy where bacteriophages exert a selection for multidrug-resistant (MDR) bacteria to become increasingly sensitive to traditional antibiotics.

Another important safety aspect to be considered in planning phage therapy is the risk of horizontal gene transfer. It is known that the constant presence of antibiotics in the environment lead to the development of resistance strategies within the bacteria because of selection pressure. Bacteria can acquire antibiotic resistance genes through HGT in the processes of conjugation, transformation or transduction. Conjugation is referred to occur in the most often trough plasmids, i.e., autonomously replicating genetic elements. When cellular DNA is transferred in closely related bacteria, a transformation takes place [59].

Transduction is defined as an introduction of foreign DNA to the bacterial cell by a virus, therefore bacteriophages and especially temperate ones may play a role in this process. Therefore, phages with a tendency to a high rate of transduction events should be excluded from therapies and it is of vast importance to choose only strictly virulent phages for a cocktail in phage therapy [60]. Nowadays, a combination of bioinformatic tools is used to define the life cycle of bacteriophages that show a potency in phage therapy for aquaculture [38,61]. On the other hand, in the review of Colavecchio et al., there is an interesting suggestion expressed that the role of temperate phages and transducing ones might be overestimated [26]. It was found that antibiotic resistance genes carried by phages did not match well in a bioinformatic analysis to proteins responsible for antibiotic resistance [62]. Further, it was reported that too little studies concerning transduction were conducted in vivo to be able to draw any conclusions about its risk [26].

### 3.3. Immune Response and Clearance from the Body

Phages are known to stimulate an immune response in the body. Once they enter into the system, they cause both specific and non-specific immune responses. An innate immune system is the first to react, with a production of phagocytes to remove bacteriophages. Then, the adaptive immune system enhances this first response with lymphocytes and antibodies. These two systems act together, preventing the attachment of viruses to bacteria, and sometimes a reduced or no therapeutic effect could be observed [42,63,64,65,66].

The fact that bacteriophage lysates may have immunomodulating effects and induce both a cellular and humoral immune response was investigated by Yun et al. [67]. They used a phage lysate of *Aeromonas hydrophila* to immunostimulate *Cyprinus carpio*. It was found that fish immunized with the phage lysate had a higher survival rate after the challenge than the fish immunized with the standard vaccine with inactivated bacterial cells. Next, Schulz et al. analyzed the influence of BAFADOR^®^—bacteriophage preparation against *Aeromonas* and *Pseudomonas*—on European eel immunity. It was reported that cellular and humoral immunity was stimulated. Moreover, a cocktail was well tolerated, and mortality was reduced in groups obtaining BAFADOR^®^. It was concluded that bacteriophages induce an immune response, hence resistance to bacterial infection, which was a desirable effect [36]. The same group studied also the effect of BAFADOR^®^ on the rainbow trout’s immune system when a mixed infection of *Aeromonas* and *Pseudomonas* was induced. As in the previous case, an immunomodulating effect was achieved and higher survival rates in the treated groups were observed [68].

However, one of the crucial safety aspects linked with immune response is that during phage therapy against Gram-negative bacteria, a great amount of bacterial lipopolisacharides (LPS) is released. Released endotoxins may cause allergic reactions and result in systemic infections in organisms [69,70]. They could lead to fever or septic shock, resulting even in the death of fish and shellfish [14]. Therefore, the preparation of phage cocktails for cultured animals should be carried out carefully and it is very important to dispose of enzymes and secondary metabolites that could be toxic for fish. On the other hand, nowadays we are able to control the level of endotoxins and remove them, if needed, to produce endotoxin-free preparations. It may be performed by several methods like chromatography, two-phase partitioning, adsorption or ultrafiltration. While choosing the method, one needs to consider the economic but also regulatory aspects, as for example, when using organic solvents, it must be proved that the residuals are removed [71]. Another solution would be to use filamentous phages or genetically engineered ones that would eradicate bacteria without lysis [26]. It is also worth to mention that no adverse side effects have been observed so far when the phage therapy was applied in aquaculture because lower vertebrates are reported to be resistant to endotoxic shock. Therefore, LPS is rather considered here as an immunostimulant, inducing an immune system to react [36,68].

Another challenge concerning phage therapy and the immune system is the difficulty in reaching the site of bacterial colonization in in vivo conditions. As described by Colavecchio et al., a key factor to the effectiveness of a phage therapy is that enough of the viruses can reach and attack the target, i.e., bacterial cells [26]. It can only be achieved if bacteriophages are effectively delivered to the site of infection and they are not cleared by the immune system of an animal (e.g., fish). It is one of the major constraints when switching from in vitro to in vivo conditions. There was a concern reported in the review by Kalatzis et al. that phage therapy in fish can cause the adaptive immune system to react, clearing phages from the body and preventing them from reaching the site of infection [47]. However, it was studied before by Nakai et al. in yellowtail and by Park et al. in ayu, that phage-neutralizing antibodies were not found [17,72]. To the authors’ knowledge, no other results reporting antibodies production after phage therapy in aquaculture are available. Moreover, this problem of immunity can be encountered not only in living animals but also in different matrices, e.g., in raw milk where certain immune factors are present, blocking the phage attachment to bacterial cells [73].

Among the possible solutions to overcome the issue of immune response is to study each case and to choose carefully the route of administration, dose, buffers and time of exposure to phages [74]. It is of vast importance to protect the phages when they enter the system. Different solutions might be considered: microencapsulation of phages, use of protective agents or appropriate buffers [42]. Another idea includes screening for phage mutants (by genetic or chemical methods), aiming at the attenuation of immunogenicity of surface proteins so that phages are not easily cleared by the immune systems of animals [75]. Further, phage cocktails composed of different bacteriophages are desirable because they could then help in phage survival in living systems besides neutralization by antibodies [42].

### 3.4. Formulation and Administration of Phage Preparations

In order for bacteriophages to be effective against bacteria, several conditions need to be fulfilled. Thus, when planning a phage therapy or use of bacteriophages as a preventive treatment, one should consider factors such as the amount of bacteriophages, time of their delivery, the availability of a bacterial host to viruses and their stability, among others. In other words, the correct application method is essential, but each biological system is different and should be considered independently. The effective use of phages in aquaculture requires also information about phage kinetics [76].

In aquaculture, ways of phage delivery include immersion, injection, delivery with feed and topical application. The most common route is the immersion of animals in phage-inoculated water [20,29,33,34,35,37,76,77,78,79,80,81,82]. Immersion is a good way for the application of bacteriophages as protective agents against bacterial diseases. However, when the disease is external or when phages cross some natural barriers and arrive at the internal site of the infection, immersion can be applied also as a treatment [68]. Khairnar described the successful usage of phages against antibiotic-resistant strains of *Pseudomonas aeruginosa* by direct application to the infected skin lesion of catfish with a cotton swab [83]. In the case of the experiments described by Park, the oral administration of phages specific to *Pseudomonas plecoglossicida* was successful even for a systemic infection of ayu fish [17].

There were several reports that compared different methods of bacteriophage administration [84,85,86]. Li et al., in 2016, published a report in which they described the use of phages to control *Vibrio cyclitrophicus* in juvenile sea cucumbers [85]. They fed phages to animals, injected phages or immersed the animals in phages. The results were compared with a control group treated with antibiotics. Feeding with a phage freeze-dried powder was as effective as the antibiotic treatment; however, it needs to be noticed that bacteriophages were given for a long time prior to infection. Feeding was more effective than immersion and the least effective was injection. Jun et al., in 2017, compared feeding and immersion, but they noticed that the dose of bacteriophages was more important than the way of administration [84].

Bacteriophages can control bacterial infections using the initial dose when it is sufficient or can replicate at the site of infection. They are able to increase their number specifically where the pathogen is located and only when the sensitive bacteria is present, allowing it to be the perfect self-dosing and self-limiting treatment at the same time [74,87,88]. However, the theory is not always true in nature, and dosing needs to be well thought out. Some reports show that the smallest applied dose was as effective or even better than the higher doses were. Li et al. stated that in the case of their experiments, the dose was not important at all because of the self-perpetuating nature of bacteriophages [16]. Wang et al. used a very small dose of bacteriophages for the immersion of abalones infected with *Vibrio harveyi*, as a higher dose would be impractical and uneconomical. The treatment was applied shortly after infection and it reduced the mortality of the animals by 70% [18]. Rong et al. tested a bacteriophage in the depuration process against *Vibrio parahaemolyticus* in oysters and achieved better results with the lowest applied phage concentration [81]. Ortega and Díaz also did not notice differences between different doses in phage therapy [80]. On the other hand, Zhang showed that the protective effect for sea cucumbers was dose dependent, and the fewer the phages, the worse the protection was against *Vibrio alginolyticus* [20]. The same effect was observed by Karunasagar, where results of bacterial reduction were clearly phage-dose-dependent [78].

Another very important factor of phage therapy is the time of administration. This parameter is highly dependent on the type of disease and degree of advance of the infection. It was shown that in the case of vibriosis, the timing of the phage addition in relation to pathogen development is crucial. In 2017, Jun et al. examined phage therapy for vibriosis. They performed three bioassays with marine shrimps infected with *V. parahaemolyticus*, that caused 100% mortality in the control groups. Phages were administered by immersion or by feeding with pellets impregnated with the phage suspension. When bacteriophages were administered 1 h after infection, 100% mortality, as in the control group, was observed. It was suggested that the disease development was extremely rapid, and bacteriophages were administered too late to help. In the second bioassay, bacteriophages were used as prophylactic agents 24 h, 6 h and 1 h before the bacterial challenge. Regardless of the method of phage administration, the survival rate was much better. The results were as follows: 50% mortality in the group fed by the phage-impregnated pellet and 25% and 50% in the groups immersed in bacteriophages. The best results were obtained in the third bioassay. Bacteriophages were administered only in the feed but they were applied both prophylactically (24 h, 6 h and 1 h before challenge) and as a treatment 1 h after the challenge. The achieved result was 0% mortality [84].

Another experiment conducted by Lomelí-Ortega and Martínez-Díaz on shrimp larvae infected with *Vibrio parahaemolyticus* showed that the timing of the treatment may be more important than the dose of bacteriophages. They were using single doses of 0.1, 1 and 10 MOI (multiplicity of infection = ratio of phage to bacteria number) and did not observe any difference between the treated groups, however, treatment 24 h post-infection had a worse effect than treatment 0 to 12 h post-infection [80].

During therapy, the activity of phages may be decreased significantly by low pH values in the gastric environment of fish, where the pH range is between 2 and 7. Parameters such as the ability of a phage to withstand gastric conditions or cross the epithelial barrier determine how they can be administrated. It may impact the degradation of phages in the gastric tract and may decrease the effectiveness of phage therapy [89]. To avoid these problems, coatings on fish feed containing phages may be applied [90]. The edible whey protein isolate coatings loaded with phages enhances the treatment of fish by reducing the loss of phage activity. Results from a simulation assay for gastric-intestinal digestion showed that this method enhances the stability of phages and reduces the level of bacteria. It allows also to control the release of phages in saltwater and protects them until they reach their destination [89,91].

Another crucial factor responsible for the success of phage therapy is an adequate selection of phages. Stalin and Srinivasan suggested that the efficiency of treatment may be improved by choosing phages with a high burst size and short lytic cycles [37]. However, there are many issues to consider while planning therapy. It is crucial to establish under controlled conditions the parameters that determine success and provide models that extrapolate from in vitro to in vivo. That is still the most challenging subject as it is hard to generalize from one in vivo situation to another [92]. Nevertheless, the consistent laboratory results concerning phage–bacterium interactions are useful to predict at least some factors that lead to successful treatment such as dose, timing and way of administration.

### 3.5. Bacteriophages Abundance and Their Stability

As bacteriophages are highly abundant in nature (total number of their particles is judged to be around 10^31^ according to most recent commentaries), it is relatively easy to isolate new virulent bacteriophages that may be potentially useful for therapy [93,94]. It is estimated that in aquatic environments, it is as much as 10^4^–10^8^ bacteriophages per ml of water [95]. Of course, not all of them are available for cultivation in the laboratory and some part of them is temperate, which makes them inappropriate for bacteriophage therapy. However, in general, searching for new phages is a rapid and cost-effective procedure. It is one of the biggest advantages over the development of antibiotics, which is a very expensive, long and complicated process. Nowadays, thanks to molecular biology and knowledge about phage biology, the time to select virulent phages useful for treatment has decreased even more [95,96]. The application of best practices allows to isolate and multiplicate phages in a simple way, and sequencing, together with novel bioinformatics tools, allow to quickly select viruses that may have potential in fighting bacterial pathogens [61].

Even though it is relatively easy to find new bacteriophages in the environment, the significant challenge for their application in commercial products is their stability [89]. The activity of bacteriophages may be influenced by several factors like the composition of the solution (presence or absence of particular ions), production process parameters (temperature, pressure) or pH of the environment. Phages are built up of proteins that may change their conformation due to harsh conditions. This may influence, for example, the structure of the phage tail and influence its ability to bind to host receptors, or even destroy the whole bacteriophage structure [96,97,98]. In the case of phage therapy when a specific dose is planned, it is very important to ensure a proper stability of phages.

Thermal and pH tolerance is crucial for the survival of phages and in consequence determines their range of application. The pH is a factor that influences the infectivity, intracellular replication and multiplication of phages. Generally, studies on the lytic activity showed that a neutral pH of 6–8 is optimum for most of the phages and their proteins, while values less than 5 and over 10 are less efficient. Less common, but also reported, are bacteriophages with another optimal pH like the ones isolated from the haloalkaline Lake Elmenteita, that have the highest infection capability at a pH between pH 10–12 [99]. High temperatures may degrade the proteins that built up the capsid. Moreover, temperature determines the viability, storage and occurrence of bacteriophages. Taking into account aquaculture waters, the temperature and pH are usually moderate and should not influence the phage activity, however, the production and formulation process parameters may not be as gentle [19,100]. Therefore, for effective production, it is better to choose phages with lower temperatures and pH sensitivities.

Several studies prove that thermal and pH stability is specific for each phage and is different depending on the phage isolate. Stalin et al., in 2017, described the *Vibrio* phages VHM1, VHM2 and VHS1 that may survive up to 50–60 °C and are tolerant to pH from 3 to 11 [37]. Another example is the *V. parahaemolyticus*-specific phage vB_VpaS_OMN isolated from oyster. This phage, with a broad host range, is temperature-tolerant up to 50 °C and has good pH stability from 5 up to 9 [19]. On the other hand, in the environment, one can mostly find more demanding phages. Nakai et al. proved that anti-*Lactococcus garvieae* phages survive in solutions of different salinity values (NaCl 0–70 g/L) but have low temperature (5–37 °C) and pH (3.5, but to 6–8) stability [101].

Another parameter important for phage stability in aquaculture water is the salt concentration. It may not influence as much the survival of phages as it influences the efficiency of phage therapy. It is reported that some bacteriophages require low concentrations of salts for the infection process, as high concentrations may induce osmotic shock, leading to the inactivation of bacteriophages. On the other hand, some level of ions is required, as they interact with capsids and stabilize the protein structure [100]. Again, stable conditions are specific to different bacteriophages. Whitman and Marshall [102] showed that psychrophilic *Pseudomonas* phages (wy and ps_1_) had low stability in high concentrations of NaCl, and Hidaka revealed that some marine phages had the highest activity in seawater [103].

### 3.6. Bacteriophage Influence on the Environment

Natural functions of bacteriophages are very important in the ecological aspects, e.g., creating bacterial diversity and supporting biogeochemical element cycling [95,104]. The virulent phages have a high degree of specificity, do not disturb beneficial bacteria and do not influence the environment [95]. It is claimed that phages are nontoxic to plants, animals and the environment, and there is little evidence of harmful phage immune responses. However, it is important to develop purified preparations, uncontaminated with bacterial components [105].

On the other hand, the eradication of a pathogen may lead to a vacated niche that can be re-invaded by other bacteria. This issue is highly debatable among scientists [106]. Phage therapy in fish farming areas may have an impact on the environment through the disruption of the microbiome. Bacteriophages regulate the number of certain bacteria in a given environment and, as a consequence, they select for some bacteria, thus changing the proportions in a community. Bacteriophages have also an important impact on the cycling of organic matter in the biosphere at a global level by releasing organic compounds through bacterial cell lysis [107]. The knowledge about these factors is especially important in the case of the aquatic environment because it allows for quick dissemination and acts as a vector for phages [91].

The probability of disruption of environmental bacterial communities may be decreased by using the smallest phages’ doses. However, phages’ pharmacokinetics is still under-explored and phages in small amounts might be below the threshold and inefficient during therapy. On the other hand, phages may reproduce and spread in the environment, not only in a targeted aquaculture system. Due to the short term experience in phage therapy, it is important to verify the impact of each phage on the microbial community before its application on an industrial scale [91,108,109].

### 3.7. Regulatory Approval and Further Perspectives

Overall, because of interesting features, bacteriophages seem to be a good choice in a future pool of antimicrobials. The scientists work on removing the technical problems connected with global use of phage therapy but the obstacles now lie in the regulations. In most countries, phages could be registered only separately, thus the registration of a phage cocktail is very difficult [91]. Up to date, there are only a few registered phage preparations worldwide and none of them are dedicated to aquaculture [26]. Moreover, these registrations were not successful in EU countries despite the growing antibiotic resistance of pathogens, which was notified by EMA. They expressed a need to fasten this time-consuming process of legislation [110]. The main problem is that the existing regulatory framework does not correspond well to phage therapy, therefore each case has to be analyzed separately [91,111]. Next, the safety of phage cocktails is still the main obstacle for their use on a mass scale, therefore more work is needed in this field including the effects of phages on the environment and the risk of gene transfer [47,91].

It seems that in this post-antibiotic era in which we are living, a final success of phage therapy is becoming more awaited than ever. The question remains in the registration path—if phages should serve as a treatment of bacterial diseases or rather as prophylactic agents. However, there are rules that may be followed to bring closer the success of designed phage therapy. The challenge is to strike the right balance between the desired and unwanted features of each phage, for example, to choose between the narrow specificity and coverage of a wide spectrum of bacteria, but the ideal phage would be strictly virulent, easily produced and stored, unable to perform transduction and free of any virulence genes. The safest application of ideal phages in aquaculture could be in closed environments such as recirculating aquaculture systems. All in all, future research should concentrate on the elimination of side effects, safety aspects and transition from the in vitro to in vivo state which would encourage a positive public opinion about phage therapy.

## Figures and Tables

**Table 1:** Benefits and challenges of different aspects of phage therapy.

Aspect of Phage Therapy	Benefit for Phage Therapy	Challenge for Phage Therapy
Specificity	Little or no interaction with natural beneficial microflora of the organism and environment	Narrow host range; not useful in case of systemic disease
Safety	Action against AMR strains; ability to adapt to changing environmental conditions (e.g., phage-resistant bacteria); biofilms destruction	Development of phage resistance; a risk of HTG; difficulty of lifecycle determination
Stimulation of immune response	Induction of immune response and resistance to bacterial infection	Clearance of phages from the organism by immune system
Formulation and administration	Self-dosing, able to replicate in in vivo conditions	A proper formulation needed to deliver bacteriophages to the site of infection
Bacteriophage abundance and stability	A lot of phages in the environment, easy to isolate, inexpensive	Sensitivity to various physico-chemical parameters resulting in instability of phages
Influence on the environment	Non-toxicity to humans, plants, animals and environment	Possibility of re-invasion of vacated niches by other bacterial species after eradication of pathogens
Regulatory/economic aspects	Growing research on phages in the entire world, a hope to replace antibiotics in a future	Still many black holes in knowledge about phages; absence of regulatory guidelines to become regular treatment

## Supplementary Materials

The following are available online: https://www.mdpi.com/2079-6382/9/6/301/s1, Table S1. Application of bacteriophages to control pathogenic bacteria in aquaculture environment.
