# Supplementary material for: Growing Trend of Fighting Infections in Aquaculture Environment—Opportunities and Challenges of Phage Therapy
Source: Antibiotics (Basel). 2020 Jun 4;9(6):301. doi: 10.3390/antibiotics9060301 (PMC7345527; doi:10.3390/antibiotics9060301)
Supplement: Supplementary file 1 [file antibiotics-09-00301-s001.pdf]

Table S1. Application of bacteriophages to control pathogenic bacteria in aquaculture environment.

	Treated aquaculture product	Bacterial pathogen	Bacteriophage, its source and way of administration	Results of phage therapy	Reference
1	Shrimp larvae (*Penaeus monodon*)	*Vibrio harveyi*	Phages were isolated from water samples from shrimp farm, hatcheries and seawater and were added directly to water	Reduced mortality of larvae	[33]
2	Shrimp larvae (*Penaeus monodon*)	*Vibrio harveyi*	Bacteriophages Viha10 and Viha8 isolated from oyster tissue and shrimp hatchery samples were added directly to water on alternative days	Reduction of bacterial biofilm; reduced mortality of larvae	[78]
3	Shrimp larvae (*Penaeus monodon*)	*Vibrio harveyi*	Bacteriophages VHM1, VHM2, and VHS1 were applied alone and in different cocktail combinations; they were isolated from water suspended sediments of the distinctive *P. monodon* shrimp aquaculture environment; phages were directly added to water	Reduced mortality of larvae	[37]
4	Abalone (*Haliotis laevigata*)	*Vibrio harveyi*	Two phages from the family *Siphoviridae* were isolated from hatchery water and oyster tissue samples; abalones were exposed to bacteriophages for 2 hours and then washed with sea water	Reduced mortality of abalone	[18]
5	Marine shrimp (*Penaeus vannamei*)	*Vibrio parahaemolyticus*	Siphoviridae phage pVp-1, isolated from the coastal water of the Yellow Sea in Korea, was fed to shrimps in form of pellets or shrimps were immersed in phage	Prophylactic activity of bacteriophages	[84]
6	Whiteleg shrimp larvae (*Litopenaeus vannamei*)	*Vibrio parahaemolyticus*	A3S and Vpms1 phages isolated from shrimp and clams cultures were directly added to water	Reduced infection and mortality of shrimp	[80]
7	Axenic brine shrimp nauplii (*Artemia franciscana*)	*Vibrio parahaemolyticus*	Vpms1 phage isolated from samples of the clam *Megapitaria squalida* collected from La Paz bay, B.C.S. México; phage was directly added to water	Prevention of vibriosis	[76]

	Treated aquaculture product	Bacterial pathogen	Bacteriophage, its source and way of administration	Results of phage therapy	Reference
8	Oyster meat	*Vibrio parahaemolyticus*	Bacteriophage OMN was isolated from Atlantic Sea water and applied to the oyster meat in presence of bacteria	Reduced bacteria concentration in oyster meat	[19]
9	Oysters (*Ostrea plicatula*)	*Vibrio parahaemolyticus*	Bacteriophage VPp1 isolated from sewage samples were added to water during depuration process	Reduced bacteria number in oysters	[81]
10	Sea cucumber (*Apostichopus japonicus*)	*Vibrio alginolyticus*	Two bacteriophages isolated from the drainpipe of an aquatic market were directly added to water	Reduced mortality of sea cucumbers	[20]
11	Prey (*Artemia salina*)	Vibrio species (*V. alginolyticus*, *V. harveyi* and *V. parahaemolyticus*)	φSt2 and φGrn1 phages were isolated from water samples collected from two locations of the north coastline of Crete, Greece; phage mixture was directly added to water	Decrease of the initial total *Vibrio* load	[35]
12	Atlantic salmon (*Salmo salar*)	*Vibrio anguillarum*	Bacteriophage CHOED isolated from bivalve samples was directly added to water	Reduced mortality of fish	[77]
13	Atlantic cod (*Gadus morhua*) and turbot (*Scophthalmus maximus*) larvae	*Vibrio anguillarum*	KVP40 bacteriophage isolated from sea water was added to eggs in presence of bacteria	Reduced and/or delayed mortality of the cod and turbot larvae	[57]
14	Zebrafish (*Danio rerio*)	*Vibrio anguillarum*	Bacteriophage isolated from sewage water from a lift station in Aveiro, Portugal was directly added to water	Reduced mortality of fish	[82]
15	Sea cucumbers (*Apostichopus japonicus*)	*Vibrio splendidus*	Three bacteriophages isolated from hatchery raw sewage (VS-1, PVS-2 and PVS-3) were fed to animals separately or in cocktail of 3 phages	Reduced mortality of sea cucumbers	[16]

	Treated aquaculture product	Bacterial pathogen	Bacteriophage, its source and way of administration	Results of phage therapy	Reference
16	Sea cucumbers (*Apostichopus japonicus*)	*Vibrio cyclitrophicus*	Phage vB_VcyS_Vc1 was isolated from hatchery raw sewage obtained from the drain-pipes; bacteriophages were administrated by feeding with phage powder, immersion or injection	Reduced mortality of sea cucumbers	[85]
17	Brook trout (*Salvelinus fontinalis*)	*Aeromonas salmonicida*	Bacteriophage HER 110 isolated from the La Petite Mouge River in France was directly added to water	Reduced mortality of fish	[34]
18	Atlantic salmon (*Salmo salar*) and rainbow trout (*Oncorhynchus mykiss*)	*Aeromonas salmonicida*	O, R and B phages, isolated at fish farms and water reservoirs in UK and France, were administered by injection, orally and by immersion	Slower rate of dying but 100% mortality was achieved as in control group	[86]
19	African sharptooth catfish (*Clarias gariepinus*)	*Pseudomonas aeruginosa*	Bacteriophage isolated from the wastewater was applied to the infected skin lesion with sterile cotton swab	Reduction of ulcerative lesion on infected fish	[83]
20	Ayu fish (*Plecoglossus altivelis*)	*Pseudomonas plecoglossicida*	One *Myoviridae* and one *Podoviridae* phages were isolated from diseased ayu and culture pond water and administered orally in feed	Reduced mortality of fish	[17]
21	Rainbow trout (*Oncorhynchus mykiss*) and zebrafish (*Danio rerio*)	*Flavobacterium columnare*	FCL-2 phage was isolated from a fish farm and was added directly to water	Reduced mortality of fish	[79]
22	European eels (*Anguilla anguilla*)	*Aeromonas hydrophila* and *Pseudomonas fluorescens*	Fish were fed with bacteriophage cocktail BAFADOR® containing 3 bacteriophages against Aeromonas hydrophila and 4 against Pseudomonas fluorescens	Stimulation of cellular and humoral immunity and reduction of mortality of the European eel	[36]
23	Rainbow trout (*Oncorhynchus mykiss*)	*Aeromonas hydrophila* and *Pseudomonas fluorescens*	Bacteriophage cocktail BAFADOR®, containing 3 bacteriophages against Aeromonas hydrophila and 4 against *Pseudomonas fluorescens*, was used for immersion or feeding of fish	Stimulation of non-specific immune system and reduction of mortality of rainbow trout	[68]

	Treated aquaculture product	Bacterial pathogen	Bacteriophage, its source and way of administration	Results of phage therapy	Reference
24	Senegalese sole (*Solea senegalensis*)	*Aeromonas salmonicida*	Phage AS-A isolated from sewage water was added directly to the water	No mortality of challenged fish, reduced number of bacteria	[29]
